# Barth Syndrome: Exploring Cardiac Metabolism with Induced Pluripotent Stem Cell-Derived Cardiomyocytes

**DOI:** 10.3390/metabo9120306

**Published:** 2019-12-17

**Authors:** Erica M. Fatica, Gina A. DeLeonibus, Alisha House, Jillian V. Kodger, Ryan W. Pearce, Rohan R. Shah, Liraz Levi, Yana Sandlers

**Affiliations:** 1 Department of Chemistry, Cleveland State University, Cleveland, OH 44115, USA; e.m.fatica@vikes.csuohio.edu (E.M.F.); g.deleonibus@vikes.csuohio.edu (G.A.D.); a.j.house@vikes.csuohio.edu (A.H.); j.kodger@vikes.csuohio.edu (J.V.K.); r.pearce52@vikes.csuohio.edu (R.W.P.); r.r.shah22@vikes.csuohio.edu (R.R.S.); 2 Cancer Center, Case Western Reserve University, Cleveland, OH 44106, USA; lxl301@case.edu

**Keywords:** Barth syndrome, induced pluripotent stem cells derived cardiomyocytes iPS-CMs, cardiac metabolism, metabolites, stable isotopes tracing

## Abstract

Barth syndrome (BTHS) is an X-linked recessive multisystem disorder caused by mutations in the TAZ gene (TAZ, G 4.5, OMIM 300394) that encodes for the acyltransferase tafazzin. This protein is highly expressed in the heart and plays a significant role in cardiolipin biosynthesis. Heart disease is the major clinical manifestation of BTHS with a high incidence in early life. Although the genetic basis of BTHS and tetralinoleoyl cardiolipin deficiency in BTHS-affected individuals are well-established, downstream metabolic changes in cardiac metabolism are still uncovered. Our study aimed to characterize TAZ-induced metabolic perturbations in the heart. Control (PGP1-TAZ^WT^) and TAZ mutant (PGP1-TAZ^517delG^) iPS-CM were incubated with ^13^C_6_-glucose and ^13^C_5-_glutamine and incorporation of ^13^C into downstream Krebs cycle intermediates was traced. Our data reveal that TAZ^517delG^ induces accumulation of cellular long chain acylcarnitines and overexpression of fatty acid binding protein (FABP4). We also demonstrate that TAZ^517delG^ induces metabolic alterations in pathways related to energy production as reflected by high glucose uptake, an increase in glycolytic lactate production and a decrease in palmitate uptake. Moreover, despite mitochondrial dysfunction, in the absence of glucose and fatty acids, TAZ^517delG^-iPS-CM can use glutamine as a carbon source to replenish the Krebs cycle.

## 1. Introduction

Barth syndrome (BTHS) is an X-linked recessive multisystem disorder associated with cardiomyopathy, neutropenia, exercise intolerance, sudden cardiac death, skeletal muscle weakness, recurrent bacterial infections and growth delay [1,2]. The approximated BTHS prevalence of 1/300,000–400,000 live births [1,3] is likely underestimated since the disorder is substantially under-diagnosed [1,4]. BTHS is caused by mutations in the TAZ gene (TAZ, G 4.5, OMIM 300394) [5], which encodes for the acyltransferase tafazzin. This protein is involved in the remodeling of tetralinoleoyl (18:2) cardiolipin, a mitochondrial membrane-associated phospholipid with a major role in mitochondrial-related processes, especially in the heart [6]. Due to the impaired ratio of monolysocardiolipin to tetralinoleoyl (18:2) cardiolipin ratio in Barth syndrome [7], mitochondria from various cell types in humans and mice demonstrate morphological abnormalities [8,9]. BTHS-affected individuals present with metabolic alterations that include lactic acidosis, 3-methylglutaconic aciduria [10], low plasma arginine [2], and a severe deficiency of mitochondrial tetralinoleoylcardiolipin [1,11]. The manifestation of cardiac disease is the major clinical feature of BTHS [12], with a high incidence in early life and, subsequently, is a leading cause of death in infants. Affected individuals can present with hypertrophic cardiomyopathy, dilated cardiomyopathy (DCM) with endocardial fibroelastosis [13], left ventricular non-compaction, ventricular arrhythmia [14], and prolonged QTc interval. There is no specific treatment for the cardiac features of BTHS, and clinical management includes supportive heart failure therapies. Thus, an understanding of the effect of TAZ mutations on heart metabolism has important implications for BTHS treatment.

Recent developments in human induced pluripotent stem cells (iPSCs) demonstrate that it is a promising model to study cellular metabolism in cardiac disorders with genetic etiology [15,16]. iPSCs are cells that have been genetically reprogrammed from adult cells back to an embryonic stem cell-like state by the forced expression of a defined set of transcription factors [17]. iPSCs are capable of generating cellullar characteristics of all three germ layers. They have the ability to proliferate and have indefinite self-renewal cycles. Cardiomyocytes derived from iPSCs recapitulate the donor genotype, reproducing the complex metabolic conditions of the affected individual’s heart and exhibit many of the characteristics of in *vivo* cardiomyocytes [18,19] including syncytial and contractile activities, ion channels, receptors, and transporters. These characteristics make iPS-CMs a good alternative model to delineate cellular mechanisms underlying BTHS cardiac phenotype.

Our study aimed to investigate the metabolic consequences of a TAZ^517delG^ mutation in an iPS-CM model of Barth syndrome. This was achieved by experimental workflows with stable isotope-labeled tracers, targeted metabolic profiling and targeted gene expression. We hypothesize that in the face of an impaired mitochondrial structure, TAZ deficiency affects cardiomyocyte energy production-related pathways and calcium homeostasis. We report our findings herein.

## 2. Results

### 2.1. Cells

**Figure 1:**
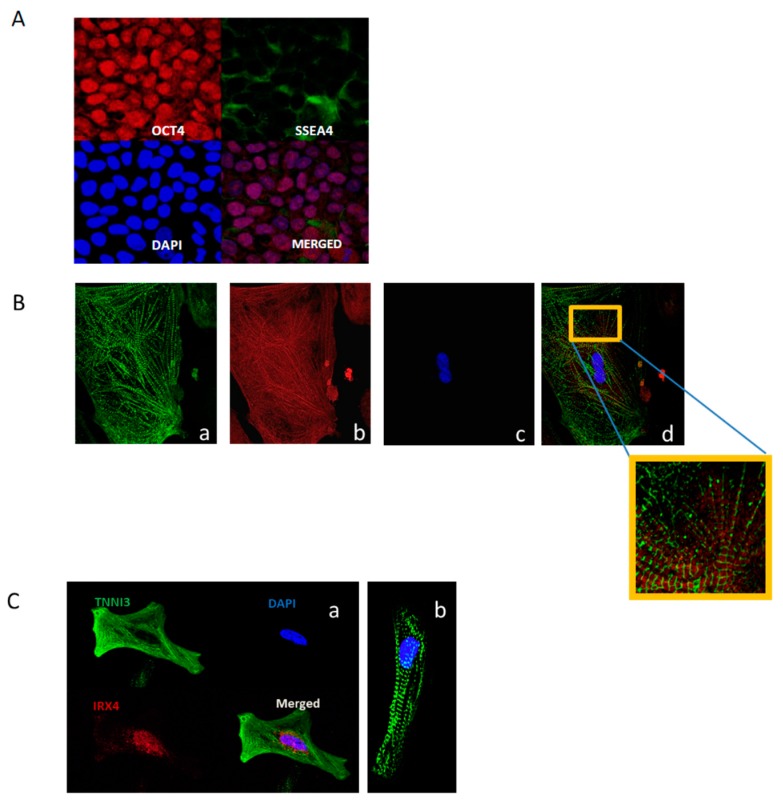
Representative images indicate successful differentiation. iPS-CM cells express cardiac specific markers (TNNI3, α-actinin and IRX4). (**A**) Immunofluorescent analysis of human iPS cells:OCT 4 (red) transcription factor expressed in undifferentiated pluripotent cells during normal development. SSEA4 is expressed on the cellular surface (green). DNA in cells nuclei is counterstained with DAPI (blue), magnification 60X. (**B**) Control-iPS-CM 45 days post differentiation. a. Sarcomeric α-actinin (green) b. TNNI3 (red), c. DNA in cell nuclei is counterstained with DAPI (blue), d. Merged panel and zoomed area, magnification 60X. (**C**) Immunofluorescent analysis of TAZ^517delG^-iPS-CM 45 days post differentiation (a). TNNI3 (green), iroquois homeobox 4 protein (IRX4) expressed in ventricular-like iPS-CM (red). DNA in cells nuclei is counterstained with DAPI (blue). (b) α-actinin (green), magnification 60X.

The iPSCs under study exhibited typical morphology and were positive for pluripotency markers OCT4 and SSEA4 (Figure 1A). Differentiated iPS-CMs at day 45 displayed sarcomeric organization as indicated by α-actinin and the appearance of *z* lines (Figure 1B) and troponin I staining (cTnI/TNNI3, the isoform expressed only in adult cardiac muscle [20] (Figure 1C). Expression of iroquois-class homeodomain protein (*IRX4)* [20] indicates the presence of a ventricular-like cell subtype (Figure 1C).

### 2.2. Carbon Substrates Preferences

**Figure 2:**
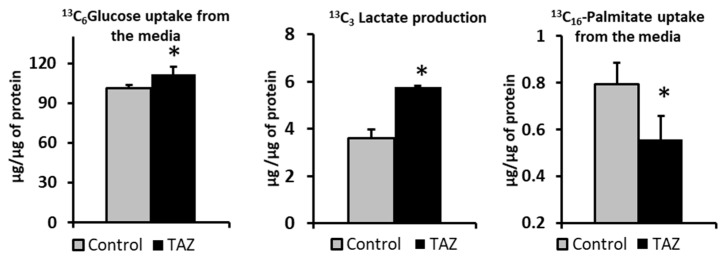
^13^C_6_-glucose and ^13^C_16_-palmitate uptake and ^13^C_3_-Lactate production after six hours. *p < 0.05. Data are expressed as the mean + SEM (*n* = 3). *p < 0.05; (*n* = 3). Error bars show a range of results from three differentiation technical replicates for each genotype. All cells were differentiated by the same protocol initiated at the same day and the experiment was performed after 45 days in culture.

Healthy adult cardiomyocytes can uptake and utilize different nutrients to support their energetic and contractile demands. The most significant portion of adenosine 5- triphosphate (ATP) synthesis in heart is driven by fatty acids catabolism; however, in response to developing pathologies, cardiomyocytes can shift their reliance from fatty acids to other carbon sources. To determine the effect of TAZ ^517delG^ on carbon source selection, control and TAZ^517delG^ - iPS-CMs were incubated first with 10 mM ^13^C_6_-glucose and 0.4 mM unlabeled palmitate (BSA conjugated) and then in a separate experiment cells were incubated with 10 mM unlabeled glucose with 0.4 mM ^13^C_16_-palmitate (BSA-conjugated). Next, we measured levels of ^13^C-labeled tracers in culture media at time zero (t = 0) and after six hours (t = 6). The comparison of the ^13^C-labeled tracers in control iPS-CM and TAZ^517delG^-iPS-CM cell media revealed that TAZ^517delG^-iPS-CMs uptake more glucose, produce more lactate, and uptake less palmitic acid (Figure 2).

### 2.3. Glucose Carbons Incorporation into Krebs Cylcle Intermediates

**Figure 3:**
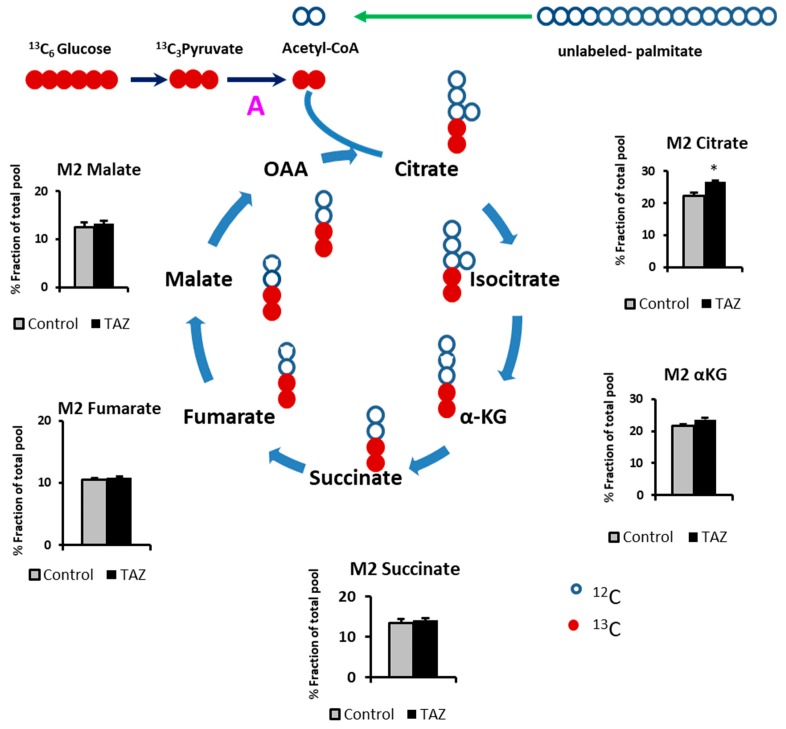
^13^C_6_-Glucose carbons incorporation into Krebs cycle intermediates. Acetyl CoA is produced through pyruvate dehydrogenase (PDH) reaction (A). Fractional contribution of ^13^C_2_-labeled isotopomers (M2). Isocitrate is under the limit of detection. Oxaloacetate (OAA) is chemically unstable. *p < 0.05. Data are expressed as the mean + SEM (*n* = 3). *p < 0.05; (*n* =3). Error bars show range of results from three differentiation technical replicates for each genotype. All cells were differentiated by the same protocol initiated at the same day and harvested at the same day after 45 days in culture.

The Krebs cycle is primary fueled by acetyl-CoA produced from glucose and fatty acids oxidation. In general, in comparison to the adult cardiomyocytes, iPS-CM cells are highly glycolytic due to their non-mature phenotype [21]. To encourage iPS-CMs to use both glucose and palmitic acid as a carbon sources, we performed all our experiments in 10 mM glucose conditions.To determine the effect of the TAZ^517delG^ on the production of Krebs cycle intermediates from glucose, we supplemented cell culture media with 10 mM labeled ^13^C_6_-glucose and 0.4 mM unlabeled palmitate (conjugated to BSA) as a carbon sources. Then, we traced ^13^C incorporation into Krebs cycle intermediates (Figure 3).

By tracking ^13^C_6_-Glucose incorporation into Krebs cycle, we observed fractional incorporation (enrichment) in most of the intermediates after six hours. The increased fraction of M2 citrate originated from ^13^C_6_-glucose indicates that TAZ^517delG^-iPS-CM has increased glucose utilization for citrate production and taken together with the decrease in palmitate uptake (Figure 2), suggests a shift towards glucose as a carbon source.

### 2.4. Evidence of Attenuated Pyruvate Anaplerosis

**Figure 4:**
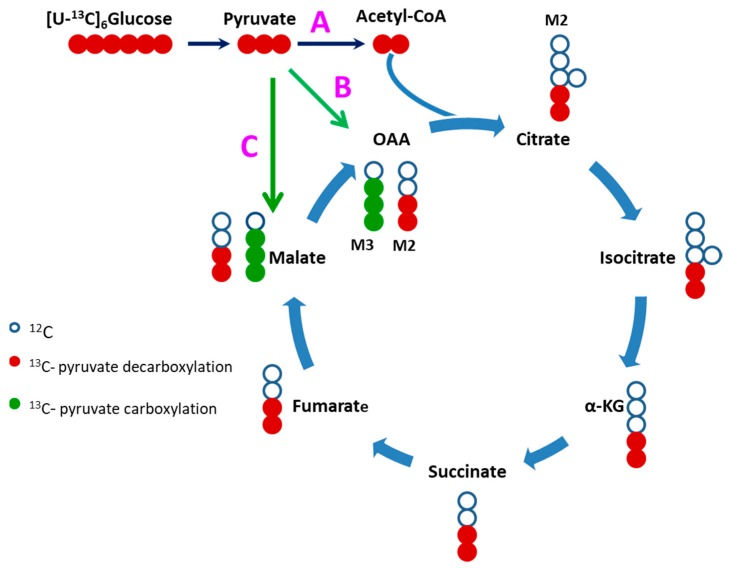
Pyruvate fates. (**A**) Pyruvate decarboxylation via pyruvate decarboxylase. Pyruvate carboxylation via pyruvate carboxylase (**B**) and malic enzyme (**C**).

Once produced from glucose, pyruvate can follow a several metabolic fates. In this experiment, we traced pyruvate fate through carboxylation by pyruvate carboxylase (PC) and malic enzyme (ME) reactions. Pyruvate carboxylation is a significant anaplerotic route that maintains a sufficient pool of Krebs cycle intermediates. ^13^C_3_-labeled malate (M3) represents pyruvate carboxylation route through pyruvate carboxylase or malic enzyme reactions (oxaloacetate is not chemically stable) while the fraction of the ^13^C_2_-labeled citrate (M2) and downstream ^13^C_2_-labeled (M2) Krebs cycle intermediates represent pyruvate decarboxylation through the pyruvate dehydrogenase (PDH) reaction (Figure 4).

**Figure 5:**
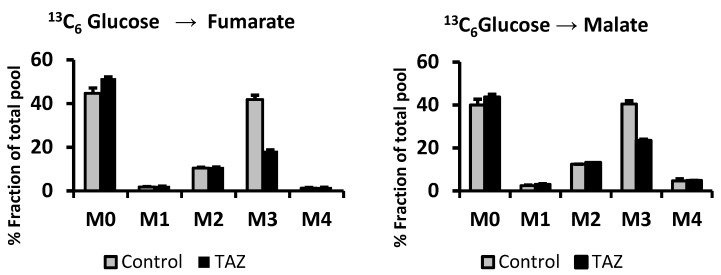
^13^C-labeled malate and ^13^C-labeled fumarate isotopomers derived from ^13^C_6_-glucose labeling. Data are expressed as the mean + SEM (*n* = 3). *p < 0.05; (*n* = 3). Error bars show range of results from four differentiation technical replicates for each genotype. All cells were differentiated by the same protocol initiated at the same day and harvested at the same day after 45 days in culture.

To assess the effect of TAZ^517delG^ mutation on pyruvate anaplerosis iPS-CMs were incubated with 10 mM ^13^C_6_-glucose for 5 h, then fractional contributions (percent of fraction) of M3 and M2 isotopomers of Krebs cycle intermediates were analyzed (Figure 4). Under our experimental conditions, the overall labeling patterns of malate and fumarate were similar (Figure 5), while ^13^C_3_-malate (M3) predominates ^13^C_2_-malate (M2) malate fractional contribution (FC (M3-malate)/FC (M2-malate) > 1) which indicates that pyruvate carboxylation favoring pyruvate decarboxylation fate in both cell lines. The difference between the ^13^C_3_-malate (M3) malate percent fraction and the ^13^C_3_-succinate (M3) percent fraction reflects the contribution of pyruvate anaplerosis to the Krebs cycle [22] (Table 1).

### 2.5. Alteration in Levels of Krebs Cycle Intermediates

**Figure 6:**
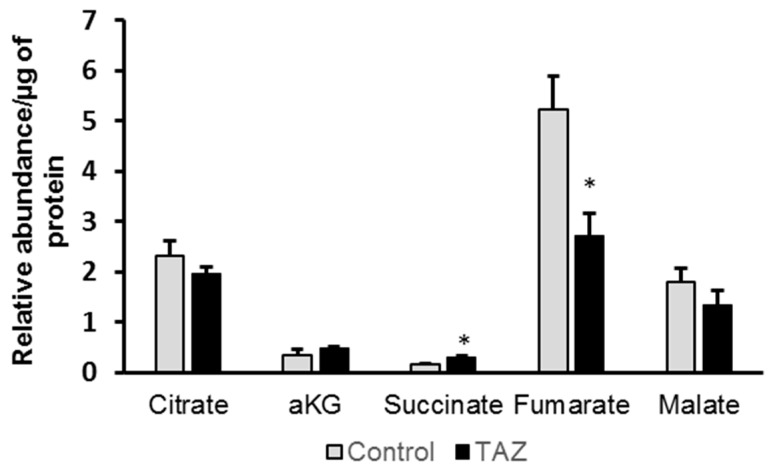
Relative abundances of Krebs cycle intermediates total pools. Relative levels are normalized to the total protein amount. Data are expressed as the mean + SEM (*n* = 3). *p < 0.05; (*n* = 3). Error bars show range of results from four differentiation technical replicates for each genotype. All cells were differentiated by the same protocol initiated at the same day and harvested at the same day after 45 days in culture.

Given the essential role of the Krebs cycle for energy metabolism, we further analyzed cellular levels of Krebs cycle intermediates and found alterations in succinate, fumarate and malate pools (Figure 6); however, only increases in succinate and fumarate were statistically significant (*p <* 0.05)

### 2.6. Alteration in Amino Acids Levels

Decreased plasma arginine and increased plasma proline are consistent findings in clinical studies among BTHS patients [11,23,24,25]. We measured levels of arginine in control and TAZ^517delG^-iPS-CMs under our standard culture conditions (RPMI 1640, 10 mM glucose) and proline under standard culture conditions and after 12 h of glucose deprivation. To compare phenotypic trends of iPS-CMs amino acid levels to patient plasma amino acid levels from the earlier published studies, fold change (FC) of arginine and proline in TAZ^517delG^-iPS-CMs or BTHS patient plasma was calculated and compared against the control cohort group (Table 2). In agreement with the clinical studies published data, TAZ^517delG^-iPS-CMs have decreased arginine and increased proline under glucose starvation in (Table 2a).

### 2.7. Disturbances in Fatty Acids Metabolism

**Figure 7:**
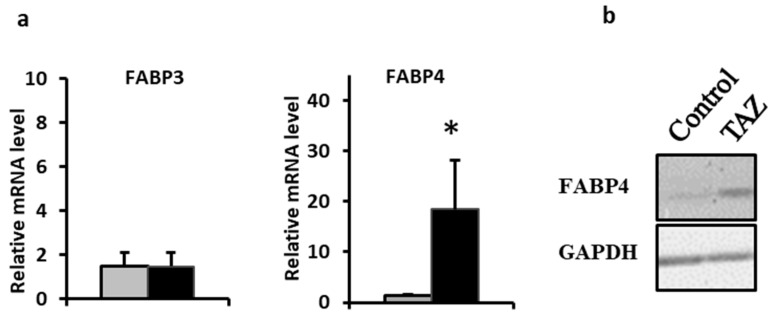
(**a**) Adipocyte fatty acid binding protein (FABP3) and heart fatty acid binding protein (FABP4) gene expression. Relative levels are normalized to the total protein amount. Data are expressed as the mean + SEM (*n* = 3). *p < 0.05; (*n* = 3). Error bars show range of results from four differentiation technical replicates for each genotype. All cells were differentiated by the same protocol initiated at the same day and harvested at the same day after 45 days in culture. (**b**) Representative heart fatty acid binding protein (FABP4) protein expression.

**Figure 8:**
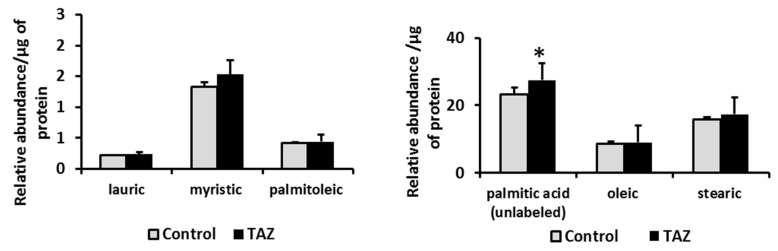
Relative levels of free fatty acids in control and TAZ^517delG^-iPS-CM by GCMS are normalized to protein amount. *p < 0.05. Relative levels are normalized to the total protein amount. Data are expressed as the mean + SEM (*n* = 3). *p < 0.05; (*n* = 3). Error bars show range of results from four differentiation technical replicates for each genotype. All cells were differentiated by the same protocol initiated at the same day and harvested at the same day after 45 days in culture.

**Figure 9:**
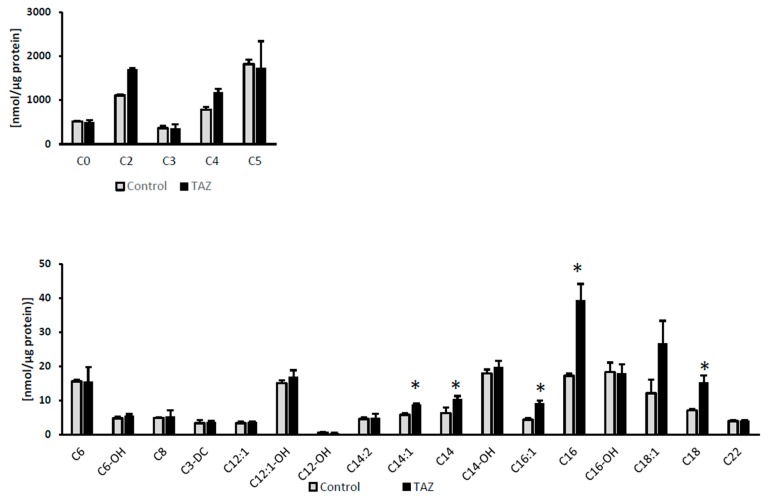
Cellular acylcarnitine levels. Relative levels are normalized to the total protein amount. Data are expressed as the mean ± SEM (*n* = 3). *p < 0.05; (*n* = 3). Error bars show range of results from three differentiation technical replicates for each genotype. All cells were differentiated by the same protocol initiated at the same day and harvested at the same day after 45 days in culture.

While fatty acids are the main carbon source for ATP synthesis in the healthy adult heart, their cellular uptake is facilitated by transporters and to a lesser extent by diffusion across the cellular membrane. Upon entrance into the cell, long chain fatty acids such as palmitate (C_16_) are rapidly activated to form acyl-CoAs in the cytosol and then are converted to acylcarnitines to enter mitochondria through the carnitine shuttle. We selectively performed a gene expression assay for a heart isoform of fatty acid binding protein (FABP3). Due to the reported association between cardiac function and levels of the adipocyte isoform (FABP4) of the protein [26,27,28,29], we also analyzed FABP4 mRNA and protein expression. While no change was found in FABP3 mRNA level, the data indicate an increase in FABP4 mRNA and the protein itself (Figure 7). Furthermore, TAZ^517delG^-iPS-CMs also exhibited an accumulation of palmitic acid (Figure 8) and of long chain acyl-carnitines (Figure 9).

### 2.8. Glucose Independent Glutamine Metabolism

**Figure 10:**
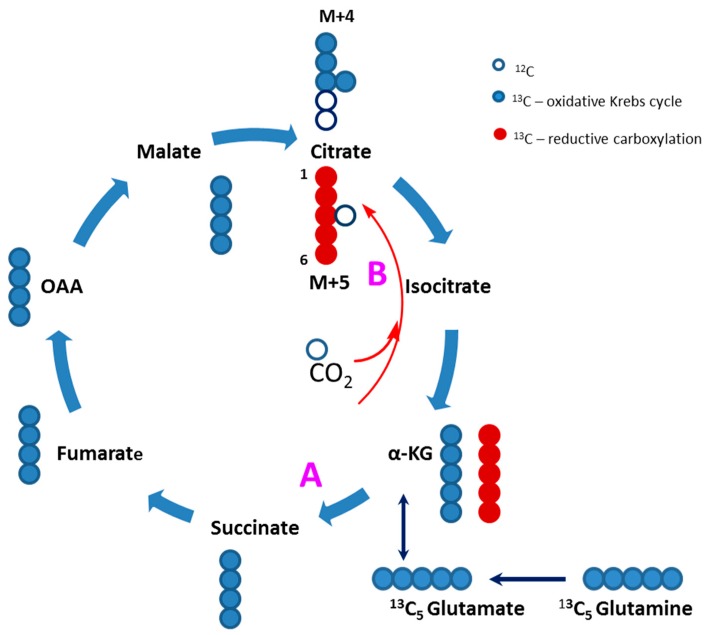
^13^C_5_-Glutamine as a carbon source for the Krebs cycle. Glutamine carbons replenish the Krebs cycle through α-ketoglutarate and proceeds through the oxidative (pathway A) and through the reductive carboxylation (pathway B) to form ^13^C_5_-labeled (M5) citrate.

**Figure 11:**
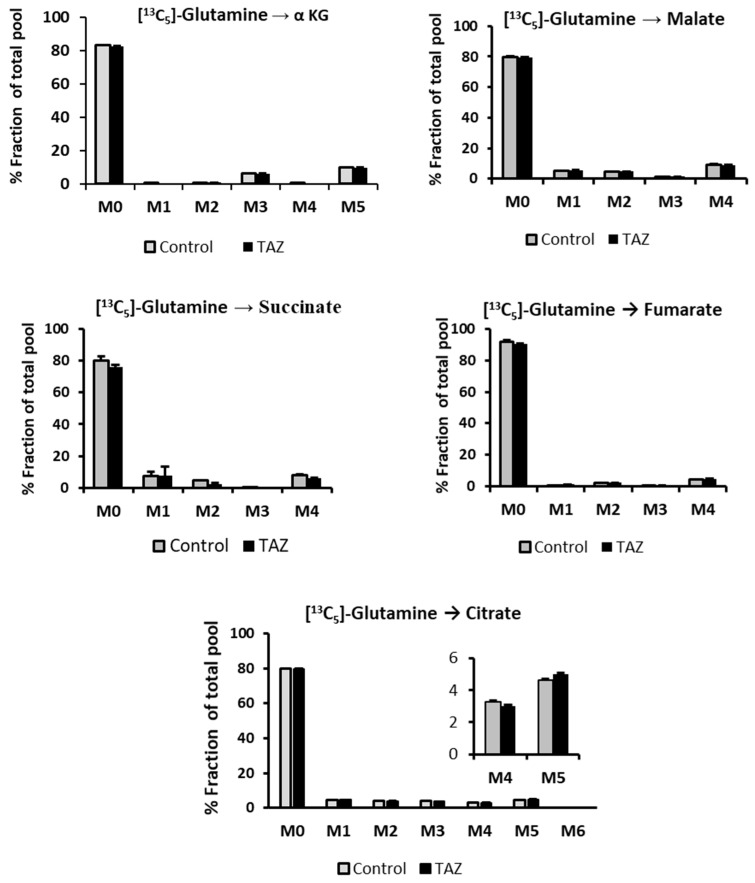
^13^C_5_-Glutamine incorporation into Krebs cycle intermediates. Relative levels normalized to the total protein amount. Data are expressed as the mean + SEM (*n* = 3). *p < 0.05; (*n* = 3). Error bars show the range of results from three differentiation technical replicates for each genotype. All cells were differentiated by the same protocol initiated at the same day and harvested at the same day after 45 days in culture.

Although fatty acids and glucose are the preferred carbon sources in the heart, glutamine is also an important fuel as it replenishes the Krebs cycle and serves as a carbon source for purine and pyrimidine biosynthesis. To determine the effect of TAZ^517delG^ on glutamine metabolism, we supplemented cells with 0.5 mM ^13^C_5_-glutamine in glucose andfatty acids depleted cell media and traced ^13^C incorporation from ^13^C_5_-glutamine to the Krebs cycle intermediates. To maintain Krebs cycle function, control and TAZ^517delG^-iPS-CM produced Krebs cycle intermediates from ^13^C_5_-glutamine. Both control and TAZ^517delG^ mutant cells produced ^13^C_4_-labeled (M4) isotopomers of citrate, fumarate and malate, indicating that ^13^C_5_-glutamine was used as an anaplerotic (replenishing) carbon source for the Krebs cycle through the conversion to the glutamate and further to α-ketoglutarate (Figure 10). However, we also found a fraction of ^13^C_5_-labeled (M5) citrate (Figure 11) as evidence that glutamine is converted to the citrate via a non-canonical reductive carboxylation (Figure 10, path B). Glutamine metabolism through the reverse action of isocitrate dehydrogenase and reductive carboxylation has been described previously as a non-significant source of citrate and lipogenic carbon in mammalian cells [30,31]. The reaction involves addition of the unlabeled carbon to ^13^C_5_-labeled (M5) α-ketoglutarate by isocitrate dehydrogenase (IDH) [32] (Figure 10).

## 3. Discussion

Since identification of TAZ gene mutations as the cause of Barth syndrome, several cellular and animal disease models have been developed. Early yeast models revealed the characteristic increase in the monolysocardiolipin to tetralinoleoylcardiolipin ratio. These cellular models facilitated the understanding of modes of tafazzin dysfunction and provided mechanistic insights regarding the phenotypic heterogeneity seen in BTHS [33,34]. Other studies involved cellular models such as fibroblasts [35], lymphoblasts [36], neutrophils [37], neonatal ventricular fibroblasts [38], and cardiac myocytes [39]. Later, mouse models were generated and revealed multiple cardiac and muscle dysfunctions [40,41] and disruption of the interactions between the electron transport chain (ETC) and some fatty acid oxidation enzymes [42].

Dysregulation of cardiac energy metabolism is recognized as a characteristic biochemical feature in various heart diseases. In this study, we investigated the metabolic consequences of a TAZ^517delG^ mutation in an iPS-CM model of Barth syndrome.

### 3.1. Lactate Production

^13^C_3_-labeled lactate (M3) is produced from the glycolytic ^13^C_3_-labeled pyruvate (M3) by lactate dehydrogenase action, thus the ^13^C_3_-labeled lactate (M3) levels measured in media represents a proxy of the cells’ glycolytic capacity. As expected for cells with defective oxidative phosphorylation, TAZ^517delG^-iPS-CMs exhibited an increased glycolytic lactate production which is essential for recycling NADH to NAD^+^ to enable continuation of glycolysis. Lactic acidosis is present in the majority of patients affected by mitochondrial diseases; however, even in the case of the severe mitochondrial dysfunction, individuals who are not under a metabolic crisis or in an immediate postprandial state may not demonstrate distinct lactate elevation. In fact, studies on Barth syndrome individuals report mildly elevated blood lactate levels, which are sometimes exercise-induced [43,44]; however, measured circulating lactate depends not only on lactate production but also on lactate consumption, thus a plasma lactate concentration does not reflect glycolytic flux but rather one static measurement.

### 3.2. Alteration in Substrate Preferences

There is emerging evidence that control of energy metabolism is a key factor in many heart diseases. In the healthy adult heart, most of the energy in the ATP form derives from fatty acids catabolism [45,46]. The metabolic flexibility of the heart allows it to shift reliance from one carbon source to another in response to developing pathologies [47,48]. We hypothesized that the TAZ^517delG^ mutation induces a carbon source shift from fatty acids to the glucose. To test this hypothesis, we performed experiments using ^13^C labeled nutrients.

First, we introduced 10 mM ^13^C_6_-glucose into cell media (depleted from unlabeled glucose) over a period of six hours to monitor glucose consumption by iPS-CMs. TAZ^517delG^-iPS-CMs consumed more glucose (101 vs. 111 µg/mg of protein respectively, Figure 2). An increase in glycolytic ^13^C_3-_lactate production (Figure 2) suggests that the observed increase in ^13^C_6_ glucose uptake in TAZ^517delG^-iPSCM is due to the higher glycolytic flux; however, based on our data we cannot verify that the main glucose transporters in heart GLUT1 and GLUT4 [49] are equally expressed in control and TAZ^517delG^-iPSCM. Mejia et al. [50] report the same expression of GLUT1 and elevated GLUT3 expression in TAZ -deficient lymphoblasts however, only GLUT1 and GLUT4 are significantly expressed in the cardiac tissue [49]. We further analyzed ^13^C incorporation in the downstream Krebs cycle intermediates. One notable finding was that the fractional contribution of ^13^C_2_-labeled citrate (M2) generated from ^13^C_6_-glucose in TAZ^517delG^-iPS-CM was found to be higher than the control (22.2% vs. 26.8%), further suggesting that TAZ^517delG^ -iPS-CMs have increased reliance on glucose as a fuel source for the Krebs cycle. Next, we introduced ^13^C_16_ -palmitate (conjugated to BSA) into the cell media. In six hours, TAZ^517delG^-iPS-CM consumed less ^13^C labeled palmitate (0.55 vs. 0.79 µg/mg of protein). While a decrease in ^13^C_16_ palmitate uptake does not provide direct evidence of a decrease in β-oxidation flux, and may also reflect a defect in fatty acids transport, the accumulation of long chain acylcarnitines (Figure 9) and the cellular total palmitate pool (Figure 8) suggest that β-oxidation is attenuated. Moreover, FABP4 overexpression indicates that that acylcarnitines transport to the mitochondria is not compromised [51]. Further investigations are required to interrogate the role of fatty acid translocase (FAT/CD36), and fatty acid transport protein (FATP) in TAZ^517delG^ induced phenotype.

Our data are in agreement with the observed increased glucose uptake in TAZ deficient lymphoblasts [50] and the most recent human study by Cade et al. who report a decrease in exercise-induced fat oxidation rate in Barth syndrome patients [52]. Our observations are also supported by a report that TazKD mice are not able to alter carbon substrate preferences from glucose to fatty acids when subjected to aerobic exercise due to the disruption between fatty acid oxidation enzymes and electron transfer chain complexes [53]. Our findings, however, are contradictory to the studies with other cellular models. Li et al. report reduced carbon flux from glucose to Krebs cycle intermediates in TAZ-KO mouse C2C12 myoblast cell line [54], while Chatzispyrou et al. demonstrate that the fractional contribution of glucose to the Krebs cycle intermediates is unaffected in TAZ-deficient skin fibroblasts [55]. The discrepancy between studies can be explained by differences in genotype and cellular metabolism for different types of cells. Wang et al., [56] report abnormalities in TAZ deficient iPSCM while same defects were not observed in primary TAZ deficient patient fibroblasts and respective iPSCs at the pluripotent stage. This fact highlights the significance of choosing a cellular model with Barth syndrome-specificphenotype.

Taken together our data suggest a metabolic shift in substrate preferences. Further studies examining control vs. TAZ^517delG^-iPS-CM responses in the presence of fatty acids oxidation and glycolytic inhibitors such as etomoxir and deoxyglucose respectively are needed to support these findings.

The substrate shift observed by us and reported by others can be attributed to metabolic adaptation toward glucose utilization. Utilization of fatty acids as a carbon source requires a proper mitochondrial function. The reliance on glucose as a carbon source improves oxygen efficiency per each generated ATP molecule. Whereas a full oxidation of one palmitate molecule requires 50 moles of atomic oxygen, a full oxidation of one molecule of glucose requires only 12 moles of oxygen [57] which makes ATP production more efficient for mitochondria dysfunctional TAZ^517delG^-iPS-CM.

The downstream consequence of the observed metabolic shift is also reflected in the accumulation of palmitic acid (Figure 8) and multiple acylcarnitines (Figure 9) which is further detrimental to the oxidative phosphorylation [58].

### 3.3. Disturbances in Krebs Cycle Intermediates

Of particular importance to cardiac energy metabolism is Krebs cycle function. A constant pool of Krebs cycle intermediates is required to produce adequate amounts of NADH and FADH_2_ under normal physiological conditions, thus there is a balance between production and efflux of all Krebs cycle intermediates. TAZ mutations induce mitochondrial dysfunction [59] and impair this balance, leading to alterations in metabolic fates. Analysis of Krebs cycle intermediates in iPS-CMs indicates a statistically significant increase in the total succinate pool in TAZ^517delG^-iPS-CMs (Figure 6). This increase may be associated with cardiolipin-dependent destabilization of respiratory chain complexes. Succinate dehydrogenase (SDH) is a component of the respiratory chain Complex II. In the presence of monolysocardiolipin (MLCL), efficient electron transfer across respiratory complexes is compromised due to loss of structural integrity [59]. Moreover, defective SDH could contribute to cardiac function by preventing full oxidation of glucose and fatty acids. In fact, Dudek et al. reported a decrease in SDH activity and protein expression levels in BTHS patient- derived iPS-CMs and cardiomyocytes isolated from a mouse model of BTHS [60]. Loss of SDH activity or expression could explain the buildup of succinate and decrease of fumarate and malate observed in our TAZ^517delG^-iPS-CM model. The decrease in malate levels was not statistically significant for *n* = 3, however, can be interpreted as a depletion of Krebs cycle intermediates due to dysregulation in the balance between anaplerotic pathways (such as pyruvate carboxylation) and the cataplerotic Krebs cycle reactions.

### 3.4. Defective Pyruvate Anaplerosis

Pyruvate is converted to acetyl-CoA through decarboxylation and enters the Krebs cycle. Alternatively pyruvate is also carboxylated to form oxaloacetate or malate via pyruvate carboxylase and malic enzyme, respectively [61]. Our isotopomer analysis reveals that TAZ^517delG^-iPS-CM have attenuated pyruvate carboxylation (Table 1); however, these data do not provide the relative contributions of each enzymatic reaction. Hence, pyruvate carboxylase and malic enzyme expression levels and activities still need to be explored. Pyruvate carboxylation is an important anaplerotic pathway in the healthy heart [62], while a disruption of pyruvate anaplerosis has been shown to cause contractile dysfunctions [63,64]. During cardiac disease pathogenesis, alterations in the balance between fatty acids oxidation and glucose oxidation may further result in dysregulation of pyruvate carboxylation. The decrease in pyruvate anaplerotic carboxylation reaction can further contribute to the contractile abnormalities [65,66].

### 3.5. Alteration in Proline and Arginine Level

A decrease in plasma arginine is a consistent finding across BTHS clinical studies It has been proposed that arginine carbons can replenish the Krebs cycle through the conversion to glutamate [11]. Accordingly, arginine supplementation has been used clinically as a dietary supplement for BTHS patients, although no studies evaluating its therapeutic effect or its role as an anaplerotic precursor have been published to date. We measured arginine by LC-MS/MS and found that arginine levels were decreased by 60% (fold of change 0.6, Table 2) in TAZ^517delG^-iPS-CMs, which is consistent with observations in patient plasma samples. We also found a significant increase in proline levels under glucose-free conditions (Table 2). In the absence of glucose, cells can use proline as a carbon source to produce ATP [67,68]. The reaction is mediated by the action of proline oxidase (POX), a mitochondrial inner membrane enzyme that interacts with respiratory complexes [68,69]. In light of TAZ-induced respiratory complexes destabilization [70], POX action can be attenuated leading to the proline increase. An increase in proline levels has been reported in several other diseases with mitochondrial dysfunction [71,72] and has been related to the lactic acidosis.

### 3.6. Glutamine as a Carbon Source

Next, we assessed the contribution of glutamine to the Krebs cycle by using a ^13^C_5_-glutamine tracer. In contrast to the adult cardiomyocytes that use fatty acids as a major carbon source, iPS-CMs strongly prefer glucose as a carbon substrate; however, in the absence of glucose or fatty acids we found that both control and TAZ^517delG^-iPS-CMs utilize glutamine to maintain Krebs cycle function. Although the observed glutamine carbons contribution to Krebs cycle intermediates is low (low enrichment), the ^13^C-labeling patterns are very similar in both control and TAZ^517delG^-iPS-CMs and the conversion of glutamine to α-ketoglutarate (through glutamate) was not significantly altered between the control and TAZ-mutant cell lines (Figure 11). This finding does not support the notion that glutamine anaplerosis through α-ketoglutarate is elevated in TAZ^517delG^-iPS-CMs. Probing cells directly with a ^13^C labeled arginine tracer is thus necessary to determine if arginine flux into the Krebs cycle is increased in TAZ^517delG^-iPS-CMs. The significance of the presented data on Figure 11 is that despite mitochondrial dysfunction, in the absence of glucose and fatty acids, TAZ^517delG^-iPS-CM can utilize glutamine as a carbon source to replenish the Krebs cycle through oxidative or reductive carboxylation pathways to the same extent as control-iPS-CM.

### 3.7. Alterations in Fatty Acids Metabolism

The cellular uptake of fatty acids is facilitated by fatty acid binding proteins (FABPs). The lipid binding protein family consists of small (~15 kDa) soluble proteins that serve as modulators of intracellular lipid homeostasis by regulating long chain fatty acid transport in the nuclear and extra-nuclear cell compartments. The FABP4 isoform is predominantly expressed in adipose tissue, but also circulates in human plasma [73,74]. It is involved in intracellular lipid trafficking and has a role in the development of insulin resistance, atherosclerosis, and inflammatory processes. FABP4 over-expression in mouse adipocytes leads to the decreased expression of mitochondrial fatty acid oxidation genes and reduced activities of mitochondrial complexes I and III [75]. Moreover, human FABP4 was shown to have a detrimental effect on rat cardiomyocyte contraction [76] *in vitro* through acutely depressed shortening amplitude and intracellular systolic peak Ca^2+^. Increased FABP4 expression in TAZ^517delG^-iPS-CM (Figure 7) suggests that FABP4 plays a role in TAZ-induced cardiac dysfunction, although the exact mechanism of FABP4 in the pathophysiology of Barth syndrome is not established yet.

Barth syndrome is manifested biochemically by low tetralinoleoyl cardiolipin content and an impaired monolysocardiolipin/tetralinoleoyl (18:2) cardiolipin (MLCL/CL_4_) ratio. The uniformly substituted (18:2) cardiolipin plays a pivotal role in the structural organization of the mitochondrial membrane [77] and is essential to acylcarnitine translocase activity [78]. Accumulation of long chain acylcarnitines observed in TAZ^517delG^-iPS-CM is in agreement with alterations in fatty acid oxidative metabolism in general [79] and supports report that cardiolipin deficiency leads to the disruption of fatty acids oxidation enzymes and electron transfer chain super complexes in TAZKDmice mitochondria [53]. The increased levels of acylcarnitines are especially striking for C14, C16, and C18 species with a fold change 1.7, 2.3, and 2.2 respectively (Figure 9) in agreement with VLCAD enzyme involvement in the *TAZ* deficient phenotype. Elevated circulating acylcarnitines levels have never been reported in Barth syndrome individuals however, we report an increase in cellular levels of acylcarnitines that may have a detrimental effect on the electrophysiology of cardiomyocytes [80,81,82,83].

In summary, our findings reveal that the *TAZ* (c.517delG) mutation induces metabolic alterations in energy production pathways and under low glucose conditions, causes a shift from fatty acids to glucose utilization as the preferred carbon substrate. In light of *TAZ* deficiency-induced impaired mitochondrial function, the increased reliance on glucose can be attributed to the improvement of oxygen efficiency per each generated ATP molecule. Whether this is a protective or maladaptive mechanism that contributes to the cardiac phenotype in Barth syndrome remains to be elucidated. Nevertheless, these results suggest that Barth syndrome patients can benefit from therapies that reduce the accumulation of toxic fatty acids catabolic intermediates. Our data also reveal that despite mitochondrial dysfunction, in the absence of glucose and fatty acids, *TAZ*^517delG^-iPS-CM can rely on glutamine as a carbon source substrate to replenish the Krebs cycle through oxidative metabolism or alternative reductive carboxylation.

Study limitations: It should be noted that more than 120 different Barth syndrome-causative *TAZ* mutations have been identified, whereas our study focused only on one specific c.517delG mutation. There are no established genotype/phenotype correlations and there is a large phenotypic variation is present within Barth syndrome affected individuals. Further studies are needed to assess metabolic and molecular changes in cells carrying different mutations in the *TAZ* gene.

## 4. Materials and Methods

### 4.1. iPS Cells

Control (PGP1-TAZ^WT^) and *TAZ* (PGP1-TAZ^517delG^) mutant cells were kindly contributed by William T. Pu, MD (Boston’s Children Hospital, Harvard Medical School). Control iPSCs were reprogrammed from healthy donor fibroblasts [79]. The isogenic (PGP1-TAZ^517delG^)-iPSC line was obtained by CRISPR/CAS9-mediated genome editing [56]. In our laboratory under an IRB-approved protocol, control iPSCs and *TAZ*^517delG^ -mutant iPSCs were proliferated to passage 25 and were cultured on 1:200 matrigel-coated plates in serum-free, chemically defined Essential 8 flex medium (Thermofisher). Prior to the differentiation, cellular pluripotency was confirmed with SSEA4 and OCT2 pluripotency markers expression.

### 4.2. Differentiation to Cardiomyocytes

**Figure 12:**
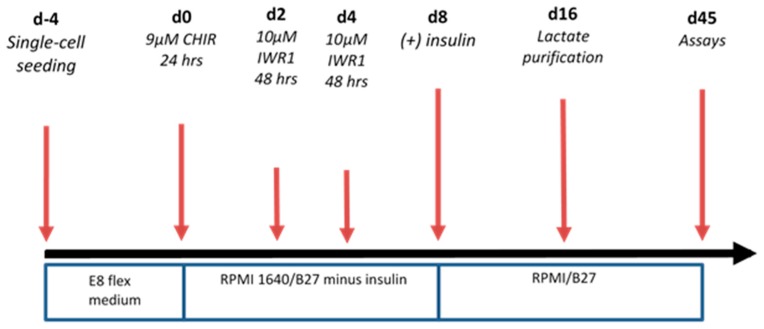
Differentiation workflow to obtain beating iPS-CM.

Differentiation was carried out through modulation of the Wnt/β catenin pathway by an optimized (Figure 12) small molecule protocol [20]

Contracting cells (videos, Supplemental materials) were first observed around day 9–14 (dependent upon initial iPSC confluency). For purification, iPS-CMs were maintained for 48 h in glucose-depleted, lactate (4 mM) enriched media [84]. To ensure cardiomyocyte maturation prior to immunostaining and metabolic studies, contracting cells were maintained in culture for 45 days. The resulting iPS-CMs are a mixture of rod-like and multi-aged cell morphologies (Figure 1).

### 4.3. Glucose Uptake and Lactate Production

To facilitate palmitic acid cell entrance, prior to experiments, palmitic acid was conjugated to bovine serum albumin (BSA) depleted from any fatty acids or lipids by charcoal purification [85].

#### 4.3.1. Glucose Uptake

On day 45, RPMI 1640 medium was aspirated from beating iPS-CMs. Cells were washed quickly with Dulbecco’s phosphate-buffered saline (DPBS) and medium was substituted with custom-prepared RPMI 1640 with 10 mM ^13^C_6_-glucose and 0.4 mM palmitic acid-BSA. 150 µL aliquots were collected at time = 0 and time = 6 h. To the 25 µL of the cell media aliquot, 10 µL of 1 mM galactose (Millipore Sigma) was added. Glucose was extracted from media aliquots using 400 µL methanol/chloroform (1:1), followed by 400 µL of water. Samples were rocked for 10 min, then centrifuged at 5000 rpm for 5 min. The resulting polar upper phase was separated from the bottom nonpolar phase and dried under nitrogen. Dried media extracts were derivatized using 0.2M hydroxylamine hydrochloride (Millipore Sigma) at 90 °C for 40 min, followed by derivatization with acetic anhydride at 90 °C for 60 min. The derivatized samples were dried down under nitrogen and re-suspended in ethyl acetate, transferred to a glass vial, and injected to GCMS. GCMS method details are found in [86] and the Figure S1.

#### 4.3.2. Lactate Production

To the 25 µL of the cell media aliquot, 10 µL of 1 mM tricarballylic acid (Millipore Sigma) was added as internal standard. Lactate was extracted from media aliquots using 400 µL methanol/chloroform (1:1), followed by 400 µL of water. Samples were rocked for 10 min, then centrifuged at 5000 rpm for 5 min. The resulting polar upper phase was separated from the bottom nonpolar phase and dried down under nitrogen. Dried media extracts were derivatized using 70 µL of N-methyl-N-tert-butyldimethylsilyltrifluoroacetamide (Millipore Sigma) at 70 °C for 40 min, transferred to a glass vial, and injected to GCMS. GCMS method details are found in the Supplemental Materials.

### 4.4. Palmitate Uptake

On day 45, RPMI 1640 medium was aspirated from beating iPS-CMs. Cells were washed quickly with Dulbecco’s phosphate-buffered saline (DPBS) and medium was substituted with custom-prepared RPMI 1640 with 10 mM glucose and 0.4 mM ^13^C_16_ -palmitic acid-BSA. 150 µL aliquots were collected at time = 0 and time = 6 h.

To the 100 µL of the cell media aliquot, 10 µL of 1 mM methyl heptadecanoate (Millipore Sigma) was added as internal standard. Palmitic acid was extracted from media aliquots using 400 µL methanol/chloroform (1:1), followed by 400 µL of water. Samples were rocked for 10 min, then centrifuged at 5000 rpm for 5 min. The resulting nonpolar bottom phase was separated from the upper polar phase, adjusted to pH 10 with NaOH, and dried down under nitrogen. Hexane (250 µL) and BF_3_/MeOH (Millipore Sigma, 250 µL) were added, then samples were incubated for 30 min at 80 °C. After derivatization, pH of samples was adjusted to pH = 3 with 1 N HCl. Saturated NaCl (150 µL) and hexane (100 µL) were added and then samples were centrifuged at 5000 rpm for 5 min. The top phase was removed, dried under nitrogen to ensure samples are free of water, re-suspended in 100 μL hexane, transferred to a glass vial, and injected to GCMS. GCMS method details are found in the Supplemental Materials.

### 4.5. Metabolic Analysis

#### 4.5.1. Krebs Cycle Intermediates

Cell media were aspirated and iPS-CMs adherent to matrigel were washed two times with cold Dulbecco’s phosphate-buffered saline (DPBS) followed by one cold water wash. Metabolism quenching was achieved by the addition of cold acetonitrile-water solution (2 mL:1.5 mL per well). Cells were scraped and lysates were transferred to tubes followed by chloroform addition (2 mL). Cell lysates were centrifuged at 5000 rpm for 15 min. The polar phase was separated and dried under a nitrogen stream at room temperature. Dried cell extracts were derivatized with methoxyamine (Millipore Sigma) in pyridine (20 mg/mL, 40 μL, 80 °C for 60 min) followed by N-methyl-N-tert-butyldimethylsilyltrifluoroacetamide (Millipore Sigma 60 μL, 70 °C for 45 min). Target ions and retention times for Krebs cycle intermediates can be found at Table S1.

#### 4.5.2. Acylcarnitine Analysis

Cells were harvested by the same protocol described for Krebs cycle intermediates preparation. 100 μL of internal standard (Cambridge Isotopes, NSK B, working solution) was added to the polar phase from cell lysates. The polar phase was then dried under a nitrogen stream at room temperature. Derivatization of acylcarnitines carried out with the addition of 60 μL of 3 N HCl/n-butanol and incubation for 45 min. at 65 °C. The derivatized samples were dried and reconstituted in mobile phase B. LCMS/MS analysis was carried out with SCIEX 5500 QTrap mass spectrometer operated in +ESI/multiple reaction monitoring scans (MRM) coupled to a Shimadzu HPLC system. The separation of acylcarnitines was achieved on XBridge BEH C18 XP column, (130 Å, 2.5 μm, 2.1 mm × 75 mm) at 0.6 mL/min flow rate with gradient program (Supplemental materials) and water/0.1% formic acid as mobile phase A and acetonitrile/0.1% formic acid as mobile phase B. LCMS/MS method details can be found in the Tables S2–S4.

#### 4.5.3. Amino Acids Analysis

Cells were harvested as described for Krebs cycle intermediates. The polar upper phase, was dried and derivatized at 70 °C for 30 min with N,O-Bis(trimethylsilyl)trifluoroacetamide (Millipore Sigma). Proline analysis was performed by GCMS operated on scan mode. Separation was achieved by 5977 GC-MS (Agilent) operated in EI/scan mode and equipped with an HP-5ms column (Agilent) 1 mM (10 µL) tricarballylic acid (Millipore Sigma) was used as internal standards. Proline fragmentation patterns were confirmed by NIST library. For arginine analysis the polar upper phase, was dried followed addition of 60 µL 3 N HCl/n-butanol (Millipore Sigma). Samples were incubated at 65 °C for 30 min, dried and reconstituted in acetonitrile /formic acid (0.1%). Arginine analysis was performed by flow injection mass spectrometry analysis (FIA-MS) with SCIEX 5500 QTrap mass spectrometer operated in +ESI/single ion monitoring mode (m/z 231-m/z 70). (House et al. in press). -D_4_-^13^C_5_-Arginine (Cambridge Isotopes, NSK A working solution) was used as internal standard (m/z 236-m/z 75).

#### 4.5.4. Free Fatty Acids Analysis

Cells were harvested as described for Krebs cycle intermediates. The nonpolar bottom phase, was dried and derivatized at 70 °C for 30 min with N,O-Bis(trimethylsilyl)trifluoroacetamide (Millipore Sigma). Retention times and fragmentation mass spectra of all fatty acids were confirmed with commercially available standards and the National Institute of Standards and Technology (NIST) library. Retention time for fatty acids can be found in Table S5.

### 4.6. Gene Expression Analysis

RNA from cells in independent biological triplicates was extracted using RNAzol (Molecular Research Center, Inc) according to the manufacturer’s instructions. RNA quality was assessed by NanoDrop. cDNA was generated using GeneAmp RNA PCR (Applied Biosystems). Real-time qPCR was carried out using TaqMan Assays-on-Demand Probe technology (Applied Biosystems). Details on probes that used for the experiment can be found in Supplemental materials. The following probes were used (Thermo-Fisher): FABP4 Hs01086177_m1, FABP3 Hs00997360_m1, SERCA2A Hs00544877_m1. 18s rRNA (4352930, Thermo-Fisher) was used as a reference gene. Relative expression levels were calculated as 2^−ΔΔCT^.

### 4.7. Calculations

#### 4.7.1. Metabolites Level

Metabolites’ levels were normalized to the internal standard (tricarballylic acid for Krebs cycle intermediates, ^13^C_3_-lactate, proline, fatty acids and corresponding stable isotope labeled standards for arginine and acylcarnitines) and then to the total protein amount of each cell pellet as measured by Bradford total protein assay. Mass spectra were used to calculate the ratio of peak areas of target metabolites to the corresponding internal standard.

#### 4.7.2. Statistical Analysis

The data were presented as the mean ± SEM from multiple samples. Significance was tested with paired two tailed *t*-test using GraphPad calculator or Student t-test. A difference of *p ≤* 0.05 was considered significant, *n* = 3 (biological replicates) for control-iPS-CM and TAZ-iPS-CM was used in most experiments, unless indicated otherwise.

#### 4.7.3. Mass Isotopomer Analysis

Mass isotopomers are molecules with the same molecular structure which differ only by the number of ^13^C atoms present, resulting in different molecular weights which can be resolved by mass spectrometry. For example, M2-Citrate signifies that two out of six carbons are labeled with ^13^C. Peak areas of each mass isotopomer were integrated from GCMS chromatograms and corrected for natural abundance of all elements contained in the analyzed molecule. The percent fraction of total pool (percent enrichment) of a given mass isotopomer (*Mi*) is expressed as the percent fraction of that specific isotopomer to the sum of all isotopomers, including the unlabeled component, M0.

(1) $$\%\;fraction\;of\;Mi\;from\;total\;pool=\frac{Mi}{\sum M0,M1,\;M2\ldots \mathrm{Mn}},$$

## Figures and Tables

**Table 1:** Fractional contributions (FC) (percent fraction of total pool) and pyruvate anaplerotic contribution. ^1^ Pyruvate anaplerosis, ^*^p < 0.05.

iPSCM	M2 Malate	M3 Malate	M3 Fumarate	FC(M3 Malate)/ FC (M2 Malate)	M3 Succinate	(FC M3 Malate)- (FC M3 Succinate) ^1^
Control-iPS-CM	12.43 ± 1.01	40.5 ± 1.4	41.8 ± 1.9	3.25	4.79 ± 0.4	35.7
TAZ^517delG^-iPS-CM	13.2 ± 0.52	23.4 ± 0.59*	18.4 ± 0.53*	2.68	3.2 ± 0.5	20.2

**Table 2:** (**a**) Arginine and proline concentrations (μM/mg protein) measured in TAZ^517delG^ and control-iPS-CMs and plasma amino acids levels published in earlier clinical studies (μmol/L). (**b**) Calculated fold changes (FC) for arginine and proline in TAZ^517delG^-iPS-CMs vs. control-iPS-CM and in BTHS affected individuals vs. healthy reveals the same phenotypic pattern.

**a**	**Arginine**			**Proline**
	Current iPS-CM study (µmol/mg protein)			
	**Control**	**TAZ**	**Control**	**TAZ** ^517delG^
Non starved	5.02	3.05	0.46	0.44
Glucose starved	NA	NA	0.15	0.45
	Plasma clinical studies [9,24] (µmol/L)			
	**Control**	**TAZ**	**Control**	**TAZ** ^517delG^
[24]	100	50	190	280
[11]	69.8	42.9	164.7	291.1
[23]	68	29	NA	NA
**b**	**Arginine**			**Proline**
	Current iPS-CM study calculated fold change (FC)			
Non starved	0.6			1
Glucose starved	NA			3
	Plasma clinical studies calculated fold change (FC)			
[24]	0.5			1.5
[11]	0.6			1.8
[23]	0.4			NA

## Supplementary Materials

The following are available online at https://www.mdpi.com/2218-1989/9/12/306/s1, Figure S1: GCMS total ion chromatogram (TIC) shows glucose/galactose separation; Table S1: Target ions and retention times for unlabeled (M0) Krebs cycle intermediates and ^13^C_3_ lactate as MTBSTFA derivatives GCMS analysis; Table S2: LC gradient program for acyl carnitines analysis;; Table S3: Acylcarnitines mass spectrometry analysis data; Table S4: Acylcarnitines retention times; Table S5: Retention times for free fatty acids as BSTFA derivatives GCMS analysis Video S1: Representative video 1 of beating iPS-CM; Video S2: Representative video 2 of beating Ips-CM.
