# Supplementary material for: Barth Syndrome: Exploring Cardiac Metabolism with Induced Pluripotent Stem Cell-Derived Cardiomyocytes
Source: Metabolites. 2019 Dec 17;9(12):306. doi: 10.3390/metabo9120306 (PMC6950123; doi:10.3390/metabo9120306)
Supplement: Supplementary file 1 [file metabolites-09-00306-s001.zip › Supplemental materials _Metabolites.pdf]

iPSCM immunostaining protocol

iPS-CMs were replated onto matrigel-coated coverslips in 6-well plates on day 21. On day 45, cells were washed twice with 1x PBS and fixed with 4% formaldehyde in PBS for 10 minutes at room temperature. Coverslips were washed once more with PBS, then maintained at 30 minutes at room temperature for permeabilization with 0.3% TritonX, and blocking with 3% goat serum in 1x PBS. Primary antibodies anti-TNNI3 (Invitrogen, PA1-86820) 1:750, IRX4 (Invitrogen, PA5-40481) 1:750, and anti-α-actinin (Invitrogen, MA1-22863) were diluted in 0.3% TritonX, 2% goat serum in 1x PBS. Cells were incubated with primary antibodies at 4°C overnight, and then were washed 3 times with 1x PBS. Next, cells were incubated with secondary antibodies CF543 (Biotium, 20308) 1:250, AlexaFluor® 555 (Invitrogen, A27017) 1:200, and AlexaFluor® 488 (Invitrogen, A11059 and A11055) 1:200 for 45 minutes at room temperature, followed by three washes with 1x PBS for five minutes. Coverslips were mounted onto microscope slides using EverBrite mounting medium with DAPI (Biotium). Cells were visualized with Nikon A1R confocal microscope.

GCMS method for glucose uptake

GCMS analysis was carried out with a 5977 GC-MS (Agilent) operated in EI mode and equipped with an HP-5ms column (Agilent). Retention times and fragmentation mass spectra of were confirmed with commercially available standards and the NIST library.

Oven program as following: initial oven temperature 80°C, hold for 3 minutes, then ramp 15°C/minute up to 190°C , ramp 5°C/minute up to 205°C, ramp 1°C/minute up to 212°C and finally ramp 15°C/minute up to 310°C hold for 2 minutes. The complex oven program is required to achieve maximum separation between structural isomers $^{13}C_6$-glucose and galactose (Figure S1). The following ions were monitored under single ion monitoring mode (SIM): m/z 319, for $^{13}C_6$-glucose, and m/z 314 for galactose.

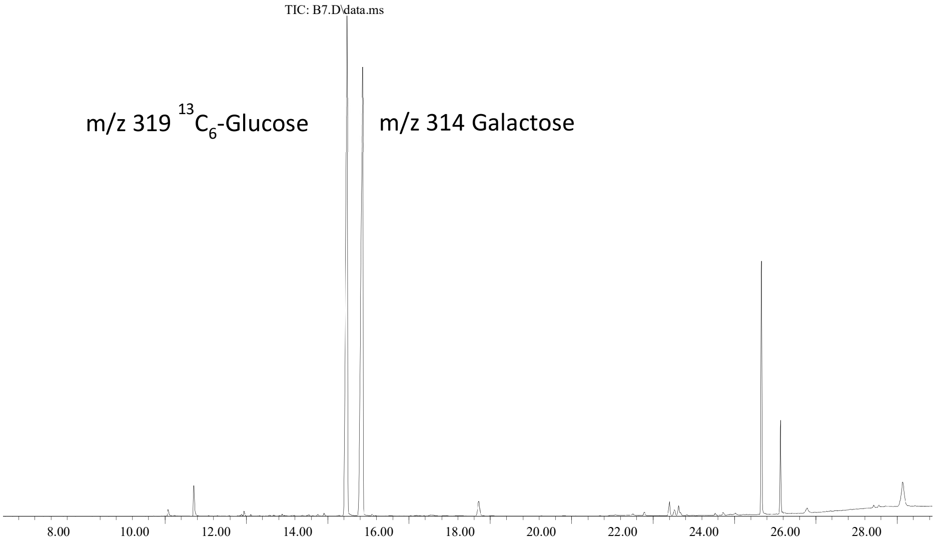

Figure S1. GCMS total ion chromatogram (TIC) shows glucose /galactose separation

GCMS method for palmitate uptake

GC-MS analysis was carried out with a 5977 GC-MS (Agilent) operated in EI mode and equipped with an HP-5ms column (Agilent). Retention times and fragmentation mass spectra of were confirmed with commercially available standards and the NIST library. Oven program as following: Initial oven temperature set at 100°C, ramp 8°C/minute up to 300°C and hold for 2 minutes. Following ions were monitored: m/z 270 for unlabeled methyl palmitate, m/z 286 for $^{13}C_{16}$-labeled methyl palmitate (retention time 14.5 min), m/z 284 for methyl heptadecanoate (retention time 16 min).

GCMS method for Krebs cycle intermediates and lactate

GC-MS analysis was carried out with a 5977 GC-MS (Agilent) operated in EI mode and equipped with an HP-5ms column (Agilent). Retention times and fragmentation mass spectra of all metabolites were confirmed with commercially available standards and the NIST library. Unlabeled (M0) ions monitored through the method are presented in Table S1. Oven program as following: initial oven temperature set at 80°C, hold for 3 minutes, then ramp 15˚C/minute up to 305°C and hold for 3 minutes.

Metabolite	m/z (SIM)	Retention time
**$^{13}C_3$-Lactate**	264	9.74
**Succinate**	289	11.89
**Fumarate**	287	12.09
**Malate**	419	14.26
**α-ketoglutarate**	346,431	13.61
**Citrate**	459	17.12
**Tricarballylic acid (internal standard)**	461	15.6

Table S1. Target ions and retention times for unlabeled (M0) Krebs cycle intermediates and $^{13}C_3$ lactate as MTBSTFA derivatives GCMS analysis

LC-MS/MS for acyl carnitines

ESI source temperature 700⁰C, ion spray voltage + 4500V. Column Waters Xbridge BEH C18, XBridge BEH C18 XP Column, 130Å, 2.5 µm, 2.1 mm x 75 mm. Mobile phase A 0.1 % formic acid in water, mobile phase B 0.1 % formic acid in acetonitrile. Injection volume 1 µl Gradient program:

Time (min)	Percent of mobile phase B
**0**	20
**1**	20
**8**	37
**22**	100
**26**	100

**26.1**	20
**30**	20

Table S2. LC gradient program for acyl carnitines analysis

ID	Q1 Mass (Da)	Q3 Mass (Da)	Dwell (msec)	DP	EP	CE	CXP
**C0**	218	85	15	100	2	20	10
**C0 ISTD**	227	85	15	100	2	20	10
**C2**	260	85	15	100	2	20	10
**C2 ISTD**	263	85	15	100	2	20	10
**C3**	274	85	15	100	2	20	10
**C3 ISTD**	277	85	15	100	2	20	10
**C4**	288	85	15	100	2	20	10
**C4 ISTD**	291	85	15	100	2	20	10
**C5**	302	85	15	100	10	20	10
**C4-OH**	304	85	15	100	2	20	10
**C5 ISTD**	311	85	15	100	10	20	10
**C6**	316	85	15	100	10	20	10
**C6-OH**	332	85	15	100	10	20	10
**C8**	344	85	15	100	8	25	10
**C8 ISTD**	347	85	15	100	8	25	10
**C3-DC**	360	85	15	100	2	20	10
**C12:1**	398	85	15	100	8	25	10
**C12**	400	85	15	100	8	25	10
**C12:1-OH**	414	85	15	100	8	25	10
**C12-OH**	416	85	15	100	8	25	10
**C14:2**	424	85	15	100	2	30	10
**C14:1**	426	85	15	100	2	30	10
**C14**	428	85	15	100	2	30	10
**C14 ISTD**	437	85	15	100	2	30	10
**C14-OH**	444	85	15	100	2	30	10
**C16:2**	452	85	15	100	2	30	10
**C16:1**	454	85	15	100	2	30	10
**C16**	456	85	15	100	2	30	10
**C16 ISTD**	459	85	15	100	2	30	10
**C16-OH**	472	85	15	100	2	30	10
**C18:2**	480	85	15	100	2	30	10
**C18:1**	482	85	15	100	2	30	10

**C18**	484	85	15	100	2	30	10
**C22**	510	85	15	100	2	30	10

Table S3. Acylcarnitines mass spectrometry analysis

Analyte Peak Name	Retention Time
**C0**	0.64
**C2**	1.18
**C3**	2.19
**C4**	3.47
**C5**	5.09
**C4-OH**	5.00
**C6**	7.27
**C6-OH**	8.77
**C8:1**	9.11
**C8**	10.34
**C3-DC**	3.02
**C4DC**	4
**C12:1**	13.43
**C12**	14.08
**C12-OH**	14
**C14:2**	15.09
**C14:1**	14.90
**C14**	15.72
**C14-OH**	15.50
**C16:1**	16.01
**C16**	17.11
**C16-OH**	15.50
**C18:2**	16.39
**C18:1**	17.30
**C18**	18.29
**C22**	21.23

Table S4. Acylcarnitines retention times.

GCMS method for free fatty acids

Separation was achieved by 5977 GC-MS (Agilent) operated in EI/scan mode and equipped with an HP-5ms column (Agilent). Retention times and fragmentation mass spectra of all metabolites were confirmed with commercially available standards and the NIST library

Free fatty acid name	Retention time
**Lauric**	27.1
**Myristic**	30.6
**Palmitoleic**	33.6
**Palmitic**	34
**Oleic**	36.9
**Stearic**	37.3

Table S5: Retention times for free fatty acids as BSTFA derivatives GCMS analysis
